# Supplementary material for: Impact of Different Anti-Hyperglycaemic Treatments on Bone Turnover Markers and Bone Mineral Density in Type 2 Diabetes Mellitus Patients: A Systematic Review and Meta-Analysis
Source: Int J Mol Sci. 2024 Jul 22;25(14):7988. doi: 10.3390/ijms25147988 (PMC11277066; doi:10.3390/ijms25147988)
Supplement: Supplementary file 1 [file ijms-25-07988-s001.zip › ijms-3110032-supplementary.pdf]

Table S1: Database search strategy and relative keywords.

Search date	Database	Search strategy	Number of papers	Filters
20/01/24	PubMed	anti-diabetic drug [Title/Abstract] OR Insulin OR Sulfonylurea OR Metformin OR DPP4 inhibitor OR GLP-1 agonist OR SGLT-2 inhibitor OR thiazolidinediones AND bone marker OR bone metabolism OR bone formation OR bone resorption OR bone mineral density OR BMD OR P1NP OR OCN OR b-ALP OR CTX OR NTX OR DEXA OR DXA OR OPG OR RANKL AND diabetes OR type-1 diabetes OR type-2 diabetes OR DM OR diabet*[Title/Abstract]	120	Randomized control trials, full free texts, time frame: 01/01/2000 to 20/01/2024
20/01/24	Cochrane	diabetes OR type-1 diabetes OR type-2 diabetes OR DM OR diabet* in Title Abstract Keyword AND bone marker OR bone metabolism OR bone formation OR bone resorption OR bone mineral density OR BMD OR P1PN OR OCN OR b-ALP OR CTX OR NTX OR DEXA OR DXA OR OPG OR RANKL in Title Abstract Keyword AND anti diabetic drug OR Insulin OR Sulfonylurea OR Metformin OR DPP4 inhibitor OR GLP-1 agonist OR SGLT-2 inhibitor OR thiazolidinediones in Title Abstract Keyword	189	Trials, full free texts, time frame: 01/01/2000 to 20/01/2024

Table S2: Detailed JADAD scores for the included studies.

Studies	Randomisation		Blinding		Fate of patients	Overall score
	Randomisation mentioned or not	Randomisation appropriate or not or not clear	Blinding mentioned or not	Blinding appropriate or not or not clear		
Ljunggren et al., 2012 [36]	1	1	1	1	1	5/5
Cai et al., 2021 [44]	1	0	1	1	1	4/5
Gallo et al., 2019 [45]	1	1	1	0	1	4/5
Grey et al., 2013 [46]	1	1	1	1	1	5/5
Hygum et al., 2020 [47]	1	0	1	1	1	4/5
Rosenstock et al., 2018 [48]	1	1	1	0	1	4/5
Henriksen et al., 2011 [49]	1	1	1	0	1	4/5
Bolinder et al., 2013 [50]	1	1	1	1	1	5/5
van Lierop et al., 2012 [51]	1	1	1	1	0	4/5
Zinman et al., 2010 [52]	1	0	1	1	1	4/5
Borges et al., 2011 [53]	1	1	1	0	1	4/5
Rubin et al., 2014 [54]	1	0	1	1	1	4/5
Nybo et al., 2011 [55]	1	1	0	0	1	3/5
Akyay et al., 2023 [56]	1	1	0	0	1	3/5
Hegazy et al., 2015 [57]	1	1	0	0	1	3/5
Kanazawa et al., 2010 [58]	1	1	0	0	1	3/5
Li et al., 2015 [59]	1	1	0	0	1	3/5
Esteghamati et al., 2015 [60]	1	1	0	0	1	3/5
Bilezikian et al., 2013 [61]	1	1	1	0	1	4/5

	Anti-diabetic drugs			Placebo				Std. mean difference
Study or Subgroup	Mean	SD	Total	Mean	SD	Total	Weight	IV, Random, 95% CI
**5.1.1 SGLT2 inhibitors**								
Bolinder 2013	1.875	0.2	91	1.195	0.19	91	16.9%	3.47 [3.01 , 3.93]
Ljunggren 2012	1.369	0.19	89	1.475	0.19	91	17.2%	-0.56 [-0.85 , -0.26]
Rosenstock 2018	1.12	0.179	411	1.15	0.184	209	17.3%	-0.17 [-0.33 , 0.00]
**Subtotal (95% CI)**			**591**			**391**	**51.4%**	**0.90 [-0.89 , 2.70]**
Heterogeneity: Tau² = 2.48; Chi² = 231.89, df = 2 (P < 0.00001); I² = 99%								
Test for overall effect: Z = 0.99 (P = 0.32)								
**5.1.2 GLP-1 receptor agonists**								
Cai 2021	1.13	0.057	38	1.05	0.04	17	16.4%	1.50 [0.86 , 2.15]
**Subtotal (95% CI)**			**38**			**17**	**16.4%**	**1.50 [0.86 , 2.15]**
Heterogeneity: Not applicable								
Test for overall effect: Z = 4.59 (P < 0.00001)								
**5.1.3 Insulin analogs**								
Cai 2021	1.17	0.07	10	1.05	0.04	17	15.2%	2.20 [1.20 , 3.21]
**Subtotal (95% CI)**			**10**			**17**	**15.2%**	**2.20 [1.20 , 3.21]**
Heterogeneity: Not applicable								
Test for overall effect: Z = 4.29 (P < 0.0001)								
**5.1.4 Thiazolidinediones**								
Grey 2014	1.3	0.18	43	1.37	0.24	43	17.0%	-0.33 [-0.75 , 0.10]
**Subtotal (95% CI)**			**43**			**43**	**17.0%**	**-0.33 [-0.75 , 0.10]**
Heterogeneity: Not applicable								
Test for overall effect: Z = 1.51 (P = 0.13)								
**Total (95% CI)**			**682**			**468**	**100.0%**	**0.99 [-0.13 , 2.11]**
Heterogeneity: Tau² = 1.90; Chi² = 270.76, df = 5 (P < 0.00001); I² = 98%								
Test for overall effect: Z = 1.72 (P = 0.08)								
Test for subgroup differences: Chi² = 35.03, df = 3 (P < 0.00001), I² = 91.4%								

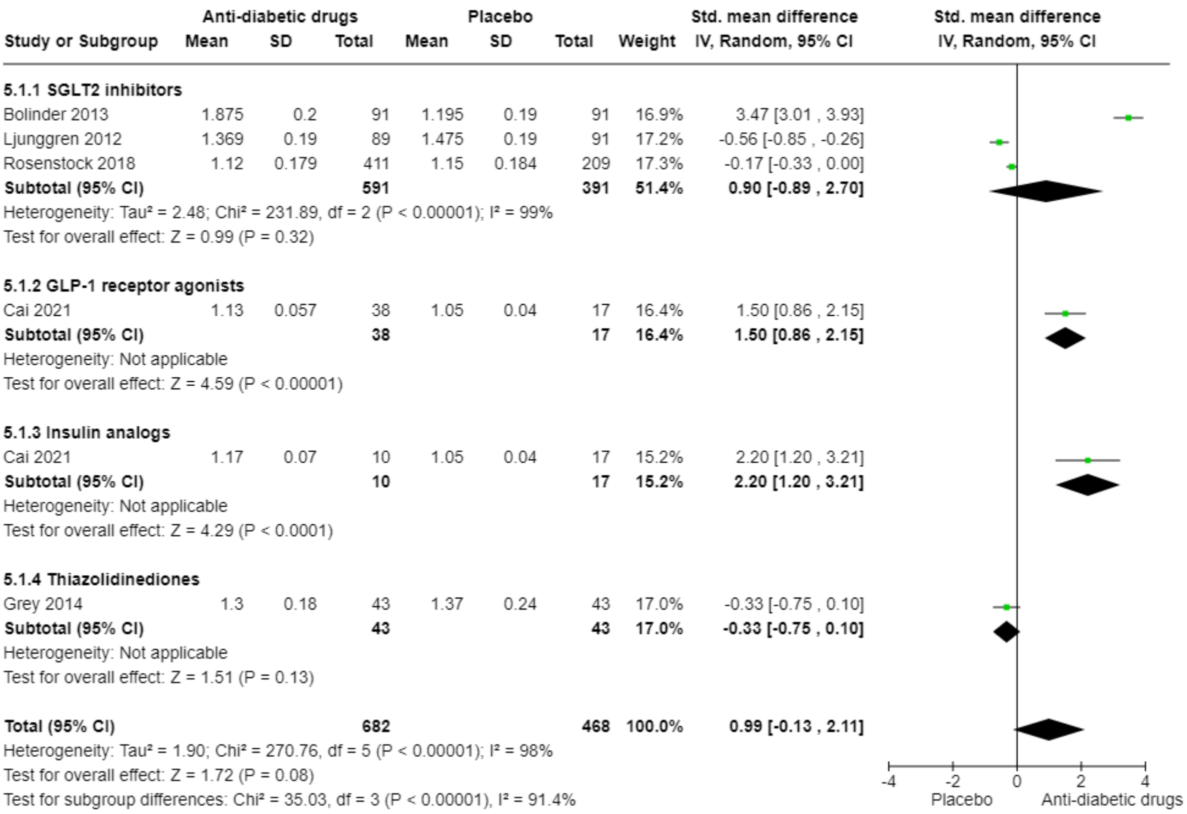

Figure S1: Forest plot for BMD of lumbar spine with subgroup analysis on the basis of class of drugs (Anti-diabetic drugs vs placebo).

	Anti-diabetic drugs			Placebo				Std. mean difference
Study or Subgroup	Mean	SD	Total	Mean	SD	Total	Weight	IV, Random, 95% CI
**5.2.1 SGLT2 inhibitors**								
Bolinder 2013	28.64	10.44	69	27.86	13.06	68	10.2%	0.07 [-0.27 , 0.40]
Gallo 2019	33.84	12.87	324	31.89	12.66	149	30.5%	0.15 [-0.04 , 0.35]
Ljunggren 2012	25.59	10.5	78	24.82	13.5	77	11.6%	0.06 [-0.25 , 0.38]
Rosenstock 2018	32.85	12.15	366	32.7	13.1	186	36.9%	0.01 [-0.16 , 0.19]
**Subtotal (95% CI)**			**837**			**480**	**89.2%**	**0.07 [-0.04 , 0.19]**
Heterogeneity: Tau² = 0.00; Chi² = 1.10, df = 3 (P = 0.78); I² = 0%								
Test for overall effect: Z = 1.25 (P = 0.21)								
**5.2.2 GLP-1 receptor agonists**								
Hygum 2020	37.3	15.3	30	37.6	15	30	4.5%	-0.02 [-0.53 , 0.49]
**Subtotal (95% CI)**			**30**			**30**	**4.5%**	**-0.02 [-0.53 , 0.49]**
Heterogeneity: Not applicable								
Test for overall effect: Z = 0.08 (P = 0.94)								
**5.2.3 Thiazolidinedione**								
Grey 2014	43.38	22	43	35	15	43	6.3%	0.44 [0.01 , 0.87]
**Subtotal (95% CI)**			**43**			**43**	**6.3%**	**0.44 [0.01 , 0.87]**
Heterogeneity: Not applicable								
Test for overall effect: Z = 2.02 (P = 0.04)								
**Total (95% CI)**			**910**			**553**	**100.0%**	**0.09 [-0.02 , 0.20]**
Heterogeneity: Tau² = 0.00; Chi² = 3.95, df = 5 (P = 0.56); I² = 0%								
Test for overall effect: Z = 1.68 (P = 0.09)								
Test for subgroup differences: Chi² = 2.85, df = 2 (P = 0.24), I² = 29.9%								

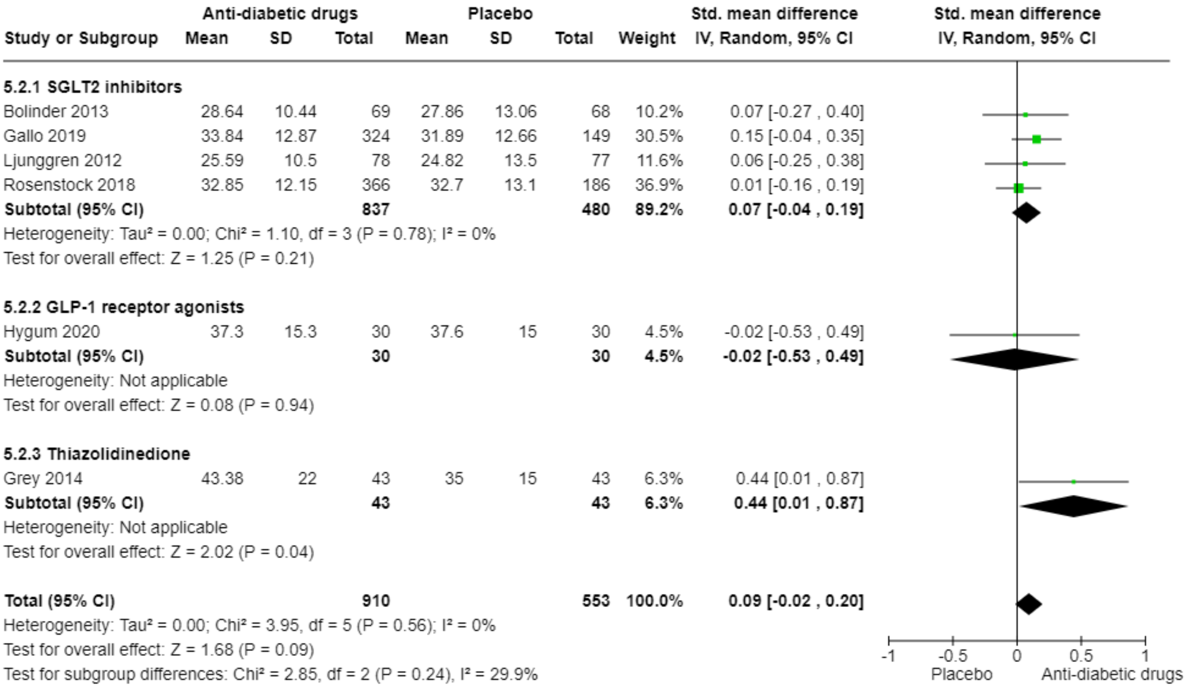

Figure S2: Forest plot for P1NP with subgroup analysis according to antidiabetic drug classes (Anti-diabetic drugs vs placebo).

	Anti-diabetic drugs			Placebo				Std. mean difference
Study or Subgroup	Mean	SD	Total	Mean	SD	Total	Weight	IV, Random, 95% CI
**5.3.1 SGLT2 inhibitors**								
Bolinder 2013	240	120	69	250	130	67	14.6%	-0.08 [-0.42 , 0.26]
Gallo 2019	322.76	154.13	319	294.92	171.51	149	24.6%	0.17 [-0.02 , 0.37]
Ljunggren 2012	260	120	78	260	130	76	15.8%	0.00 [-0.32 , 0.32]
Rosenstock 2018	336.75	161.41	359	280.6	150.4	185	26.1%	0.36 [0.18 , 0.53]
**Subtotal (95% CI)**			**825**			**477**	**81.1%**	**0.15 [-0.04 , 0.34]**
Heterogeneity: Tau² = 0.02; Chi² = 7.17, df = 3 (P = 0.07); I² = 58%								
Test for overall effect: Z = 1.57 (P = 0.12)								
**5.3.2 GLP-1 receptor agonists**								
Hygum 2020	310	44	30	320	71	30	8.2%	-0.17 [-0.67 , 0.34]
**Subtotal (95% CI)**			**30**			**30**	**8.2%**	**-0.17 [-0.67 , 0.34]**
Heterogeneity: Not applicable								
Test for overall effect: Z = 0.65 (P = 0.52)								
**5.3.3 Thiazolidinediones**								
Grey 2014	283.4	130	43	240	120	43	10.7%	0.34 [-0.08 , 0.77]
**Subtotal (95% CI)**			**43**			**43**	**10.7%**	**0.34 [-0.08 , 0.77]**
Heterogeneity: Not applicable								
Test for overall effect: Z = 1.58 (P = 0.11)								
**Total (95% CI)**			**898**			**550**	**100.0%**	**0.15 [-0.02 , 0.31]**
Heterogeneity: Tau² = 0.02; Chi² = 9.58, df = 5 (P = 0.09); I² = 48%								
Test for overall effect: Z = 1.75 (P = 0.08)								
Test for subgroup differences: Chi² = 2.30, df = 2 (P = 0.32), I² = 12.9%								

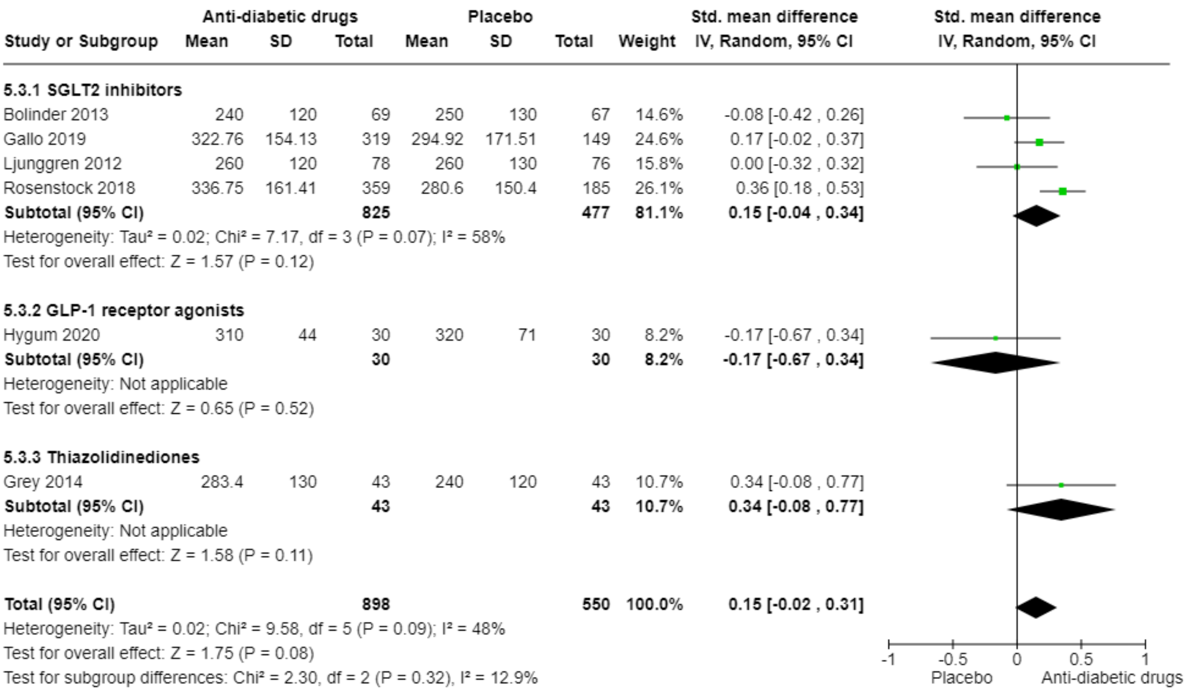

Figure S3: Forest plot for CTX with subgroup analysis according to antidiabetic drug classes (Anti-diabetic drugs vs placebo).
